# Renovascular Mistaken as Essential Hypertension due to Giant Hepatic Hydatid: A Rare Treatable Entity

**DOI:** 10.1155/2018/9614874

**Published:** 2018-03-13

**Authors:** Awaji Qasim Alnami, Liaqat Ali Khan, Said Samir Qeshta, Abdulrahman Alnami, Akram Awad, Waleed Mohalwi, Musa Tumaihi

**Affiliations:** Department of General and Laparoscopic Surgery, Sabya General Hospital, Jazan, Saudi Arabia

## Abstract

Renovascular hypertension is an unusual but treatable cause of refractory hypertension that is infrequently caused by external compression of the renal vasculature by a giant hydatid cyst, a parasitic infection, caused by *Echinococcus granulosus* in its larval stage which is endemic in many parts of the world including Saudi Arabia. The disease can produce a cyst in almost every part of the body with the liver and lungs being the most frequently targeted organs producing a variety of symptoms depending upon the site and size of the cyst. We report a case of giant hepatic hydatid cyst with the possibility of renovascular hypertension due to mass effect as evident by dramatic drop of the blood pressure to its normotensive state postoperatively.

## 1. Introduction

Renovascular hypertension is an unusual but treatable cause of refractory hypertension that is infrequently caused by compression of the renal vasculature by a giant hydatid cyst. Cystic echinococcosis (CE) is a parasitic illness caused by infection with *Echinococcus granulosus(E. granulosus)* in its larval stage [1]. The tapeworm stage is harbored in the intestine of carnivores such as dogs, which constitute the definitive host [2], and the eggs are passed in the feces of the infected carnivores and ingested by herbivores such as sheep, which comprise the intermediate host. Humans are the incidental intermediate host.

Larvae emerge from the eggs in the intestine, and after invasion to the blood vessels, they can migrate into almost every part of the body [3]. The most common site is the liver.

Large cysts, today fairly rare even in endemic areas, are called giant hydatid cysts (GHCs) [4, 5]. Hepatic hydatid cysts may show growth either in the liver capsule, gastrohepatic ligament, or peritoneum. Giant cyst as seen in our patient enlarging through the peritoneum may cause rare symptoms because of the intra-abdominal mass effect.

## 2. Case Presentation

A 24-year-old native Arabian lady, mother of four, from the high mountainous area who was managed by a local primary healthcare centre as a case of essential hypertension, was referred to the medical outpatient department of our secondary care facility for uncontrolled hypertension for the last few months. After primary assessment, the medical team put her on antihypertensive medication and sent back home. On the next visit, her BP remained high despite combined antihypertensives with ACE inhibitor, beta-blocker, and Ca-channel blocker and complaining of right hypochondrial pain, for which ultrasonography was requested along with basic laboratory workup.

U/S shows a large cyst in the liver for which the patient was referred to surgical OPD for consultation. The surgical team decided for elective surgical exploration.

On admission, the patient vitals were stable except high BP of 155/91 mmHg and high ALP (221 U/l).

**Figure 1:**
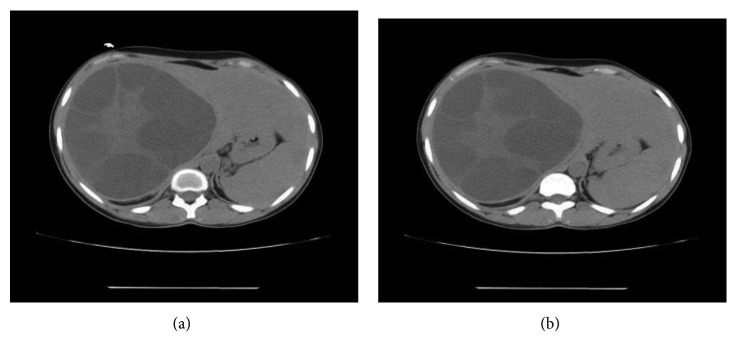
Noncontrast CT scan showing a giant cyst of the liver.

**Figure 2:**
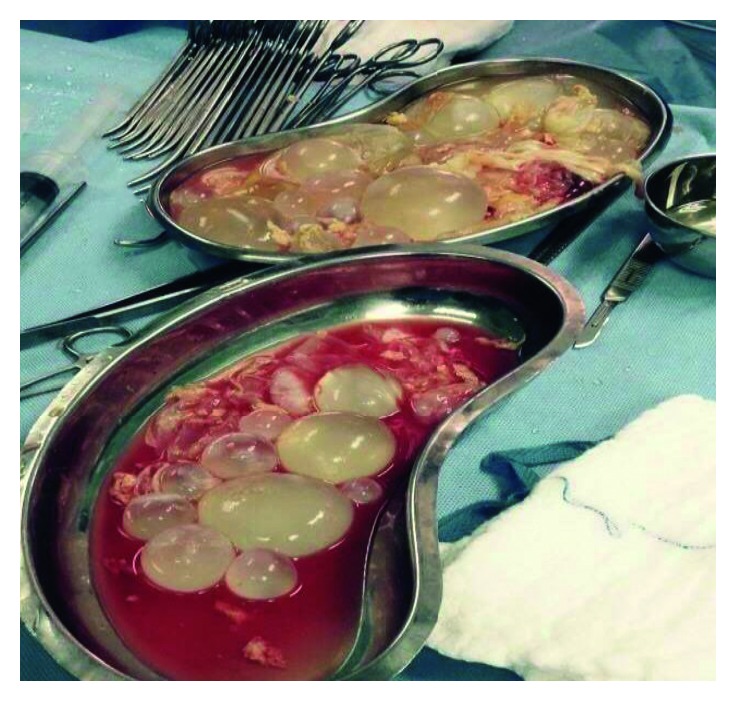
Multiple cysts postoperatively.

Ultrasonography showed a maternal right liver lobe hydatid cyst measuring 14 × 16 cm, and CT scan revealed a large sizable hypodense cystic lesion of about 150 × 150 × 200 mm in AP × TS × CC dimensions, respectively, with multiple septations inside, being mainly in the right lobe, with peripheral calcific wall seen laterally with normal liver parenchyma (Figure 1), occupying almost the whole right lobe of the liver, exerting a mass effect in the form of right kidney, right renal vasculature, and IVC compression with no invasion in addition to medial displacement of the duodenum and midline structures. Exploratory laparotomy was done, and evacuation of a large hydatid cyst with deroofing was done by the standard protocol (Figure 2). Postoperatively, the patient had a spike of high BP in the postoperative holding area, which was managed by the medical team. On the 1st postoperative day, the patient was recovering well with BP findings of 130/80 mmHg for which antihypertensive medication was withhold on the 2nd postoperative day. The patient discharged on the 8th postoperative day, and on subsequent visit to the surgical OPD, the patient was doing well with normotensive findings without antihypertensive medications.

## 3. Discussion

Most patients with a hydatid cyst in the liver are asymptomatic, and its presence becomes evident only when the liver is found to be enlarged or a cystic lesion is noted when the liver is imaged for any other reason [6]. In the general literature, arterial hypertension caused by hydatid disease has been reported only in few cases in which the cysts were located in the adrenal glands. Escudero et al. [7] reported a hydatid cyst located in the left adrenal gland which has caused the left kidney to displace inferiorly, resulting in hypertension. Tazi et al. [5] reported a giant primary adrenal hydatid cyst presenting with arterial hypertension in a 64-year-old Moroccan man who presented with the unusual symptom of arterial hypertension associated with left flank pain. Albarak et al. reported the first case of huge hepatic hydatid causing renovascular hypertension by compressing and rotating the right kidney and its vasculature in an already nephrectomized patient. Michael et al. [8] reported a case of renovascular hypertension, for a new-onset hypertensive crisis in a 54-year-old lady who presented to emergency department in whom a large benign liver cyst caused compression of the renal vasculature, and to the best of our knowledge, this is the 3rd case of hepatic hydatid disease causing hypertension. The attainment of the normotensive state after surgical removal of the cyst not only clarified the aetiology of hypertension but also provided with the management as well.

## 4. Conclusion

In conclusion, the mass effect of a giant hepatic hydatid cyst should be kept in mind as one of the unusual causes of hypertension when dealing with the patient where hydatid disease is endemic and the patient is refractory to antihypertensive medications.
